# Supplementary material for: Mesoscale neuronal granular trial variability in vivo illustrated by nonlinear recurrent network in silico
Source: Nat Commun. 2024 Nov 15;15:9894. doi: 10.1038/s41467-024-54346-3 (PMC11567969; doi:10.1038/s41467-024-54346-3)
Supplement: Supplementary file 1 — Supplementary Information [file 41467_2024_54346_MOESM1_ESM.pdf]

Supplementary Information:

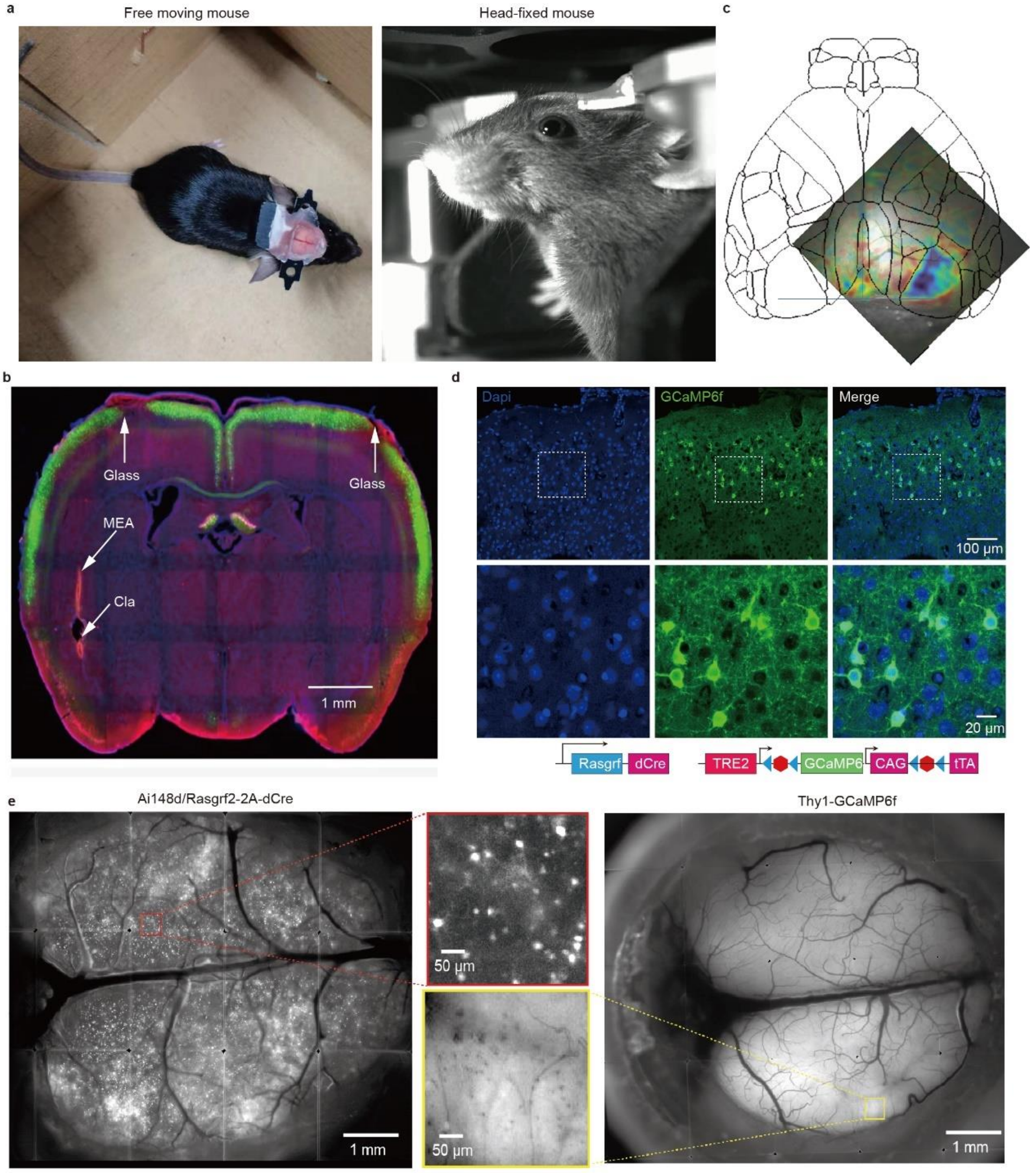

Supplementary Fig. 1 | Simultaneous mesoscale imaging and deep brain probe implantation in mice. a, Mouse adaptation and recording setup. Left : A mouse freely moving post-surgery; Right : A mouse head-fixed during habituation for mesoscale recording. **b**, Mouse brain slice (Ai148d/ Rasgrf2-2A-dCre) highlighting GCaMP6f expression in layer 2/3, the pathway of micro electrical probe array (MEA) and mesoscale imaging boundary. **c,** Retinotopic map of visual cortex in an example mouse. The borders of area V1 is determined by retinotopic mapping and cranial anatomical landmarks. The map result is similar in the related experimental mouse (n=15 mice). **d**, Transgenic strategy (bottom) to drive sparse GCaMP expression (green; top) in layer 2/3. **e**, Comparative imaging results in the transgenic mouse lines of Ai148d/Rasgrf2-2A-dCre (left) and Thy1-GCaMP6f (right).

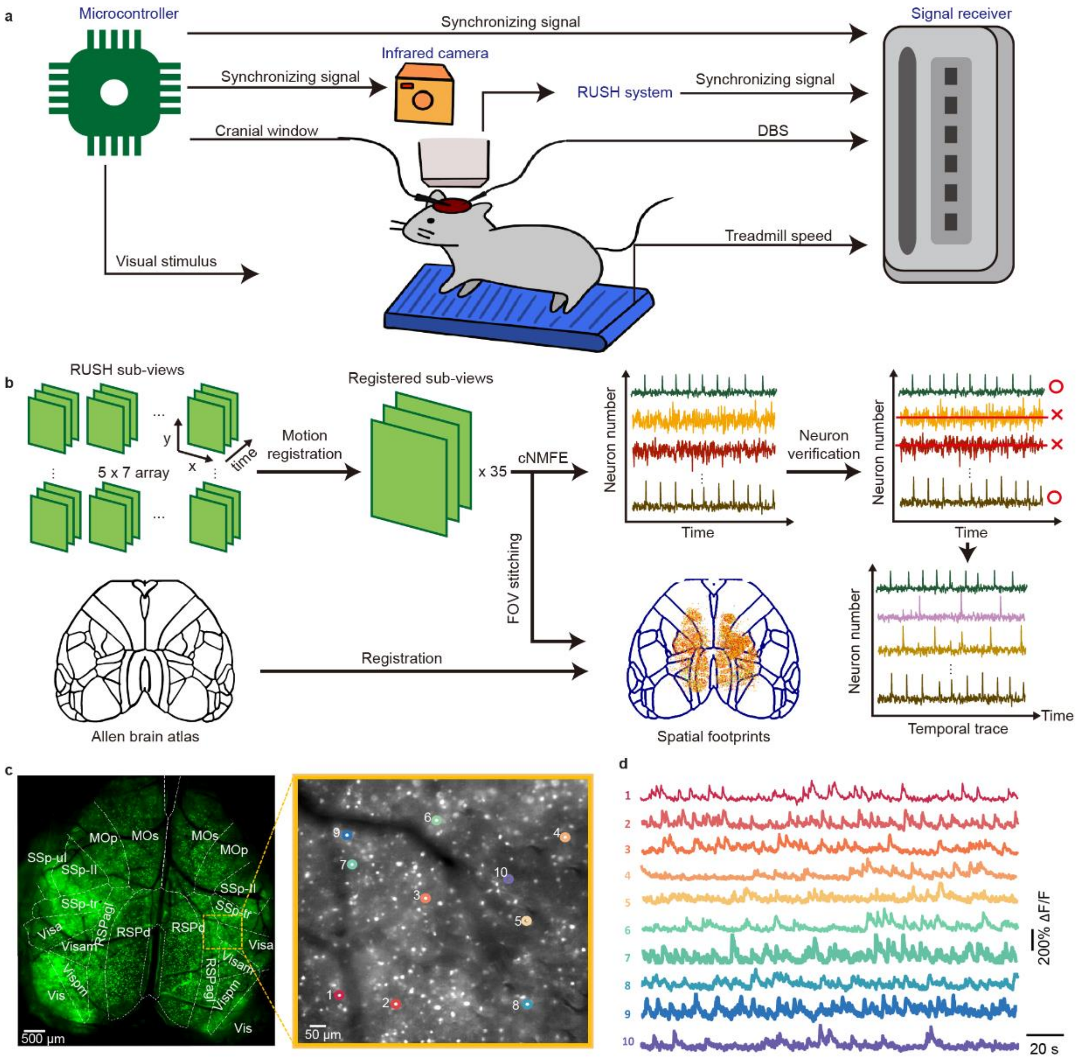

Supplementary Fig. 2 | The recording system and data pre-processing framework. **a,** Multimodal recording setup, including facial expression, running speed, cortical neural activity during experiment involving electrical or visual stimulus. **b**, Pipeline for neuron extraction from RUSH raw data, leading to obtain the spatial distributed maps and the temporal trace for all recorded cortical neurons. **c,** Neuronal imaging result. Left: A maximum projection image from a representative mouse. Right: Magnified view of the area enclosed in orange in the left panel. **d,** Z-scored traces of fluorescence calcium activity for 10 example neurons identified in panel **c**.

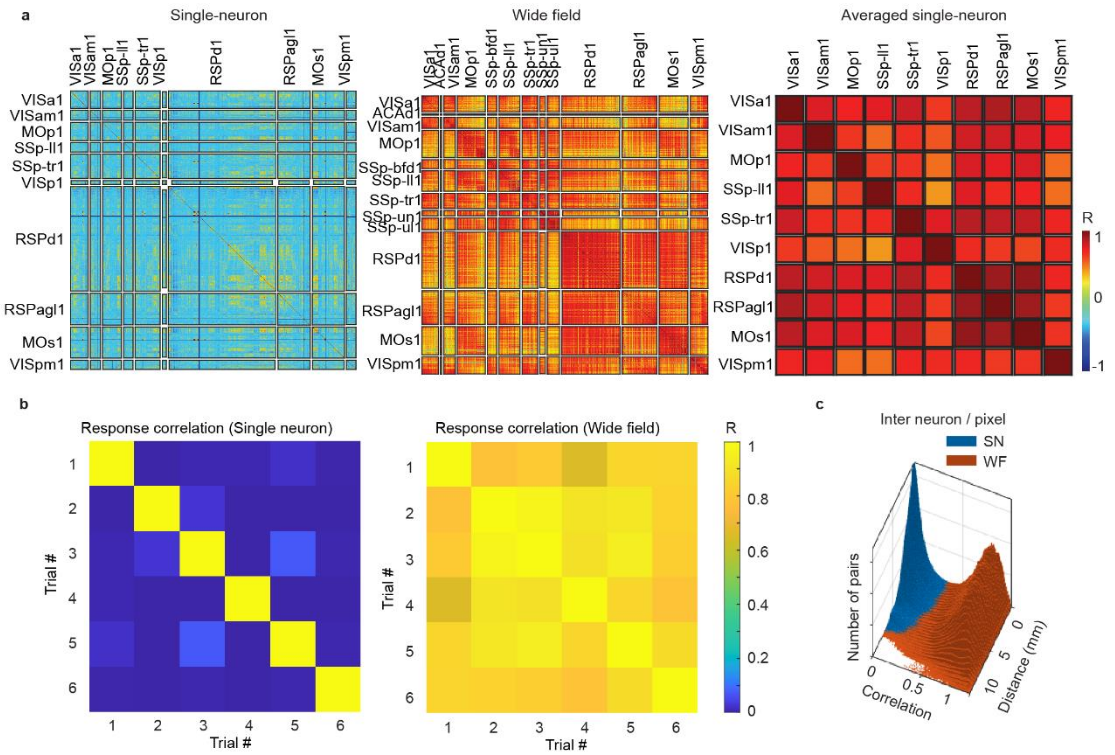

Supplementary Fig. 3 | Comparative analysis of neural correlations at single-neuron level and wide field level. a, Demonstrates spontaneous neural activity correlations across individual brain regions. Left: Correlations at the single-neuron level; Middle: Correlations at the wide-field level; Right: Correlations obtained by averaging neural activity from the single-neuron level, highlighting their similarity. **b**, Analysis of neural response consistency across trials during electrical stimulation. The left panel presents correlations at the single-neuron level, while the right panel shows correlations at the wide-field level, facilitating a direct comparison between the granularity levels of neural data. **c**, Comparison of inter-neural (single neuron) and inter-pixel (wide filed) correlation distribution of neural (pixel) pairs ranging specific distance on the cortex.

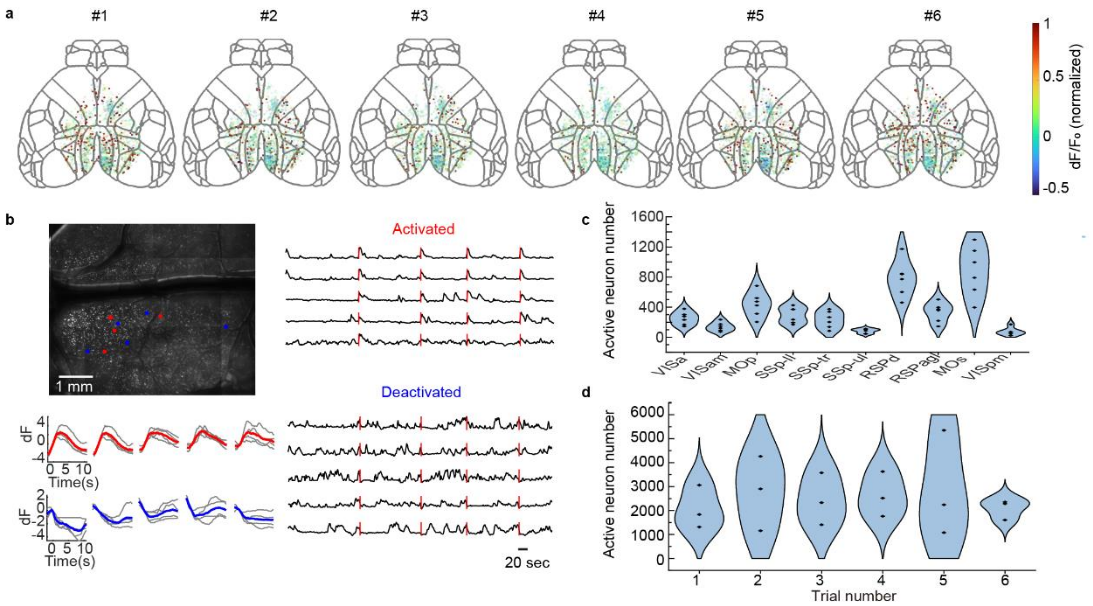

Supplementary Fig. 4 | Cortical neuron response to repeated electrical stimulus. a, Each map visualizes the intensity of responses from individual neurons across the cortex during electrical stimulation, illustrating the widespread impact of the stimulus. **b**, The activated neurons (red) and deactivated (blue)) trace shows opposite response during electrical stimulus. **c**, Violin plots of activated neuron counts across different regions over six trials from one mouse. (n=6 trials) **d**, Violin plots of total activated neuron number observed across each trial during electrical stimulus, based on data from 3 mice. (n=3 mice)

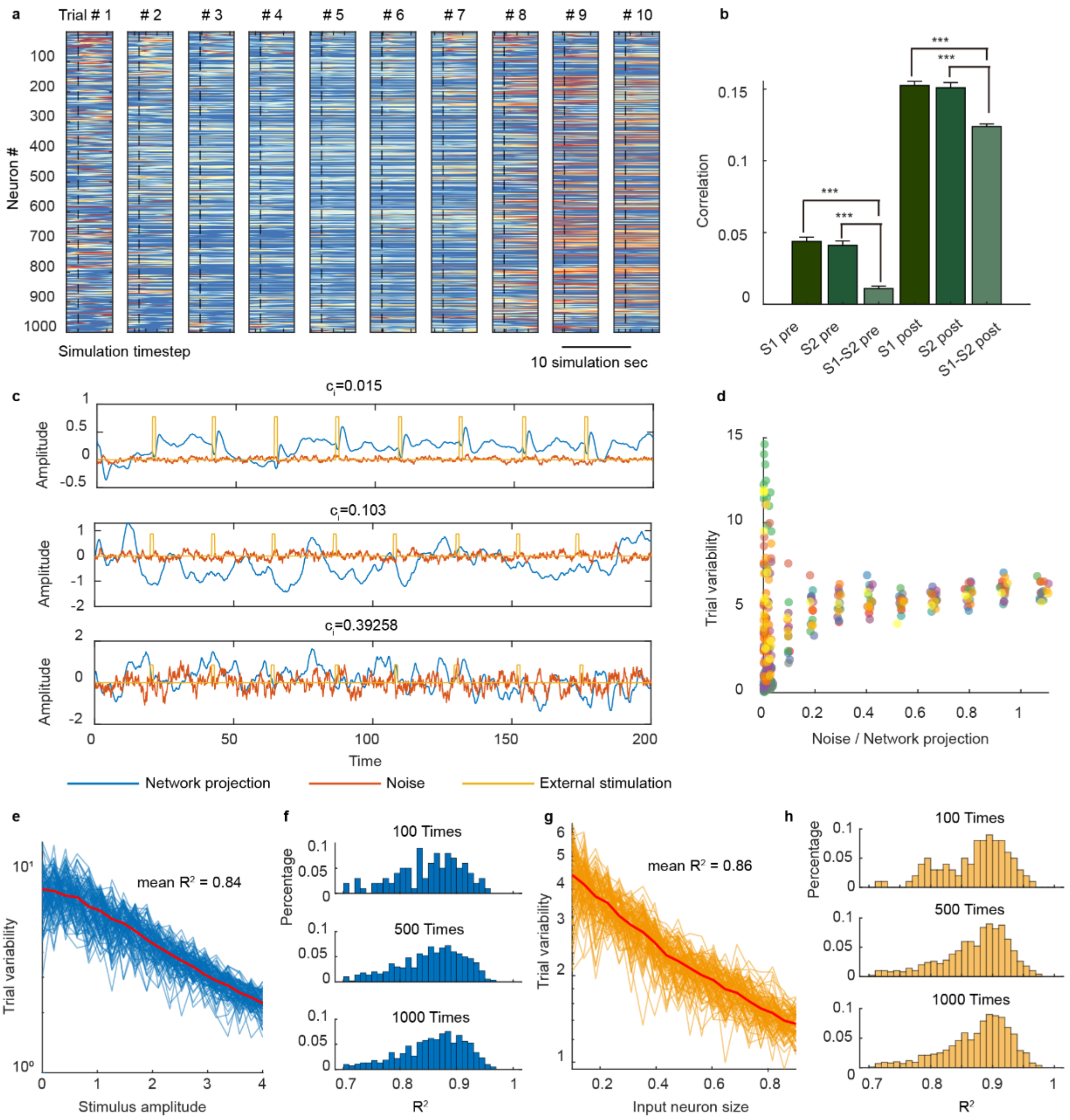

Supplementary Fig. 5 | Trial variability analysis *in silico*. a, The response of simulated neuron population, with noise parameter equals zero. **b**, The model simulated pre- and post-stimulation inter-trial mean correlation. Inner-state trials correlation and inter-state trials correlation are compared, n=100 repeats, *** for p<0.0005, error bars for SEM. **c,** Example neurons showing the component of network projection, noise and external stimulation, with increased noise level. **d,** We calculated the noise input contribution $c_i = \frac{\sum_{t=1}^{T} H_i^{wn}(t)^2}{\sum_{t=1}^{T}\left[\sum_{j=1}^{N} J_{ji}\phi\left(x_j(t)\right)\right]^2}$ of each neuron at each time point. $\langle c_i \rangle$ is the averaged value among all neurons. The plot shows the relationship between the $\langle c_i \rangle$ (noise / network projection) and the trial variability. **e**, The relationship between stimulus amplitude and trial variance, with randomly initialized connection matrix. Each blue line is a random initialization of connection, the averaged result is shown in

red solid line. **f**, The distribution of R^2 of linear fitting between amplitude and log trial variability, after 100, 500 and 1000 initializations. **g,h**, same as **e,f**, but showing the relationship between input neuron size and trial variability. Altogether, the simulation reveals that the variability decreasing is robust of random connectivity.

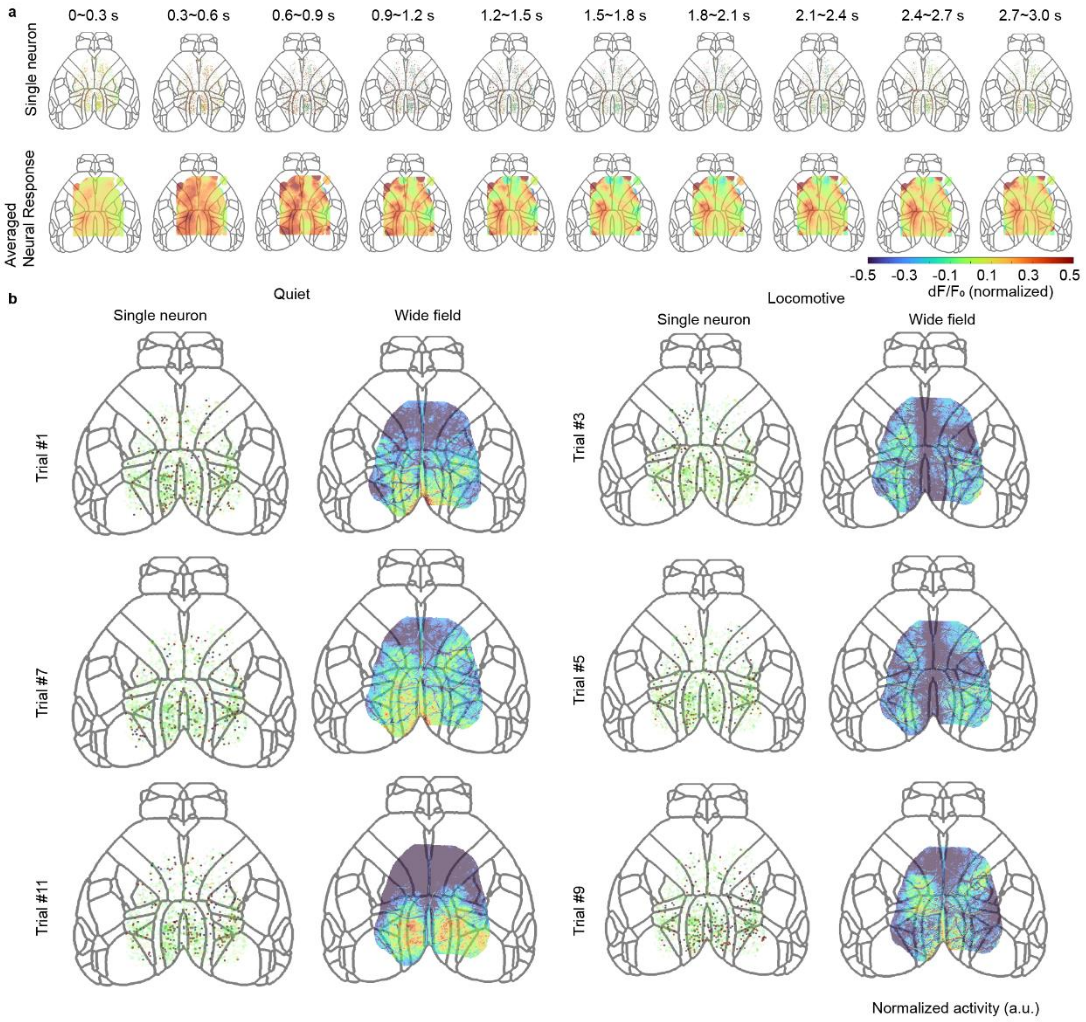

Supplementary Fig. 6 | Analysis of neuronal response following electrical stimulation. a, Top panel shows the intensity of neuronal responses immediately after stimulation onset. The bottom panel illustrates the average neural activity post-stimulation. **b**, Demonstrates cortex-wide response patterns to identical stimulation under different behavioral states – quiet (left panel) and locomotive (right panel). This comparison, made at both the single-neuron and wide-field imaging scales.

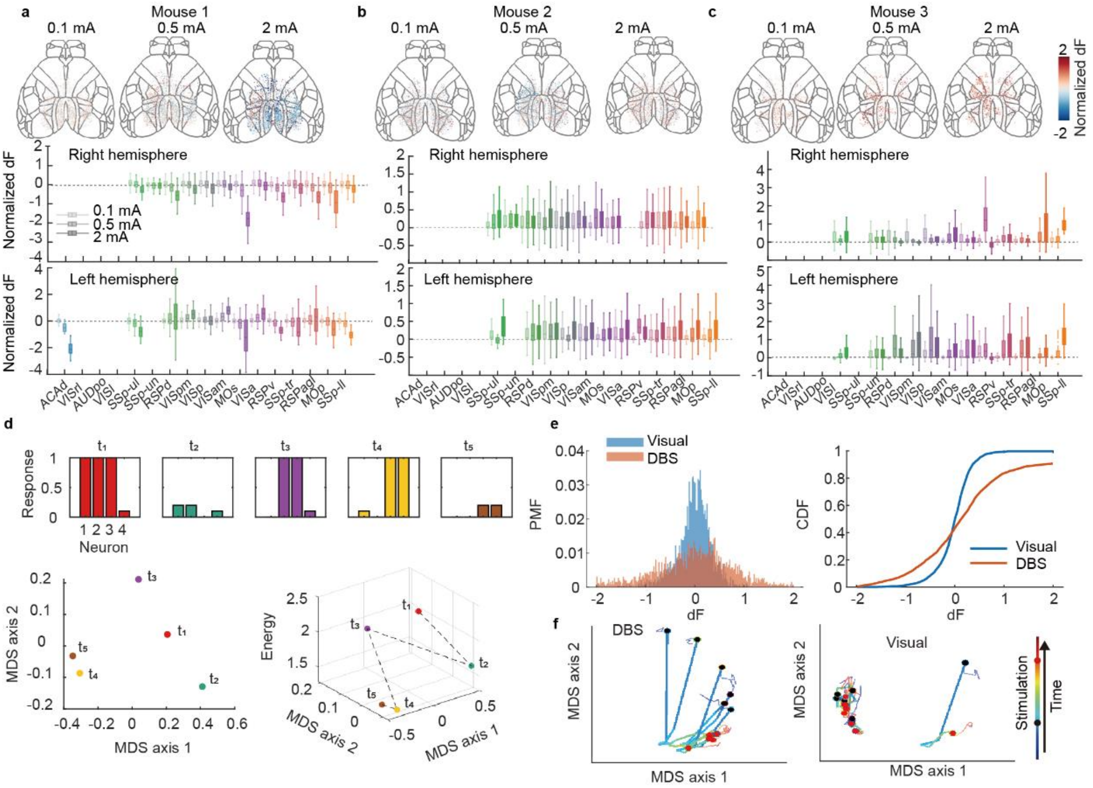

Supplementary Fig. 7 | Spatial distribution of DBS responses and comparison between DBS and visual stimulation. The neural responses under parameterized nuclear stimulation. **a, b, c** shows three mice respectively. In each panel, top shows the response of neurons with the increase of stimulation amplitude. The populational responses in each brain area are shown in right hemisphere (middle) and left hemisphere (bottom). The box indicates the range from the bottom quartile to the upper quartile. The centered line shows the median. The whiskers show the maximum and the minimum of data. n number is specific for the regional neuron number which is shown in Source Data. **d**, A simple example illustrating the principal of energy landscape. In this case, a 4-dimensional neural space at 5 distinct time points is shown. Note that although t_1, t_3 and t_4 recruits more neurons, they end up far from each other in MDS axis, because they recruit distinct groups of neurons. And although t_5 and t_4 has distinct response magnitude, because their close populational correlation, the energy is low at these 2 states, which means the system reaches a more stable state at this timepoint. **e**, The trial-averaged neural post-stimulation responses, showing the empirical probability mass function (PMF) and the cumulative distribution function (CDF). **f**, Brain state trajectory in the first 2 multidimensional scaling (MDS) axis, comparing the DBS with the visual stimulation.

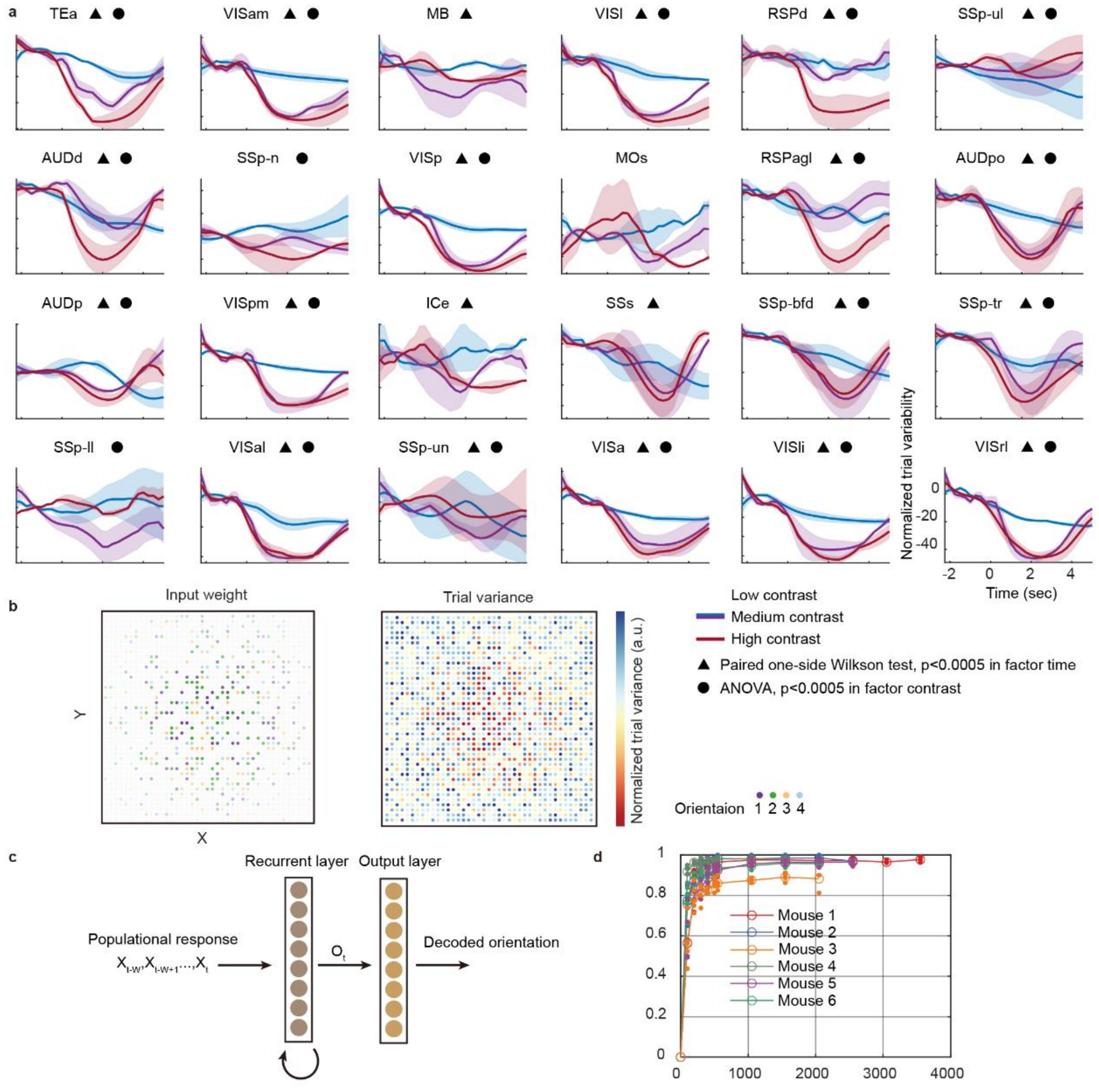

Supplementary Fig.8 | Neurons in different regions respond to visual stimulus at different contrast. a, Time-dependent mean trial variance in different cortical regions during delivery of grating videos with different contrast, shading stands for SEM, n=3 mice. **b**, The distribution of spatial heterogenous input neurons. Each color stands for a certain orientation input, higher opacity means higher input amplitude. **c,** The structure of the recurrent classification network used to decode the grating orientation. We took 2 sec response after the onset of the stimulation as the network input. **d**, Decoding accuracy in each of 6 mice, using neurons randomly sampled from left visual cortex.

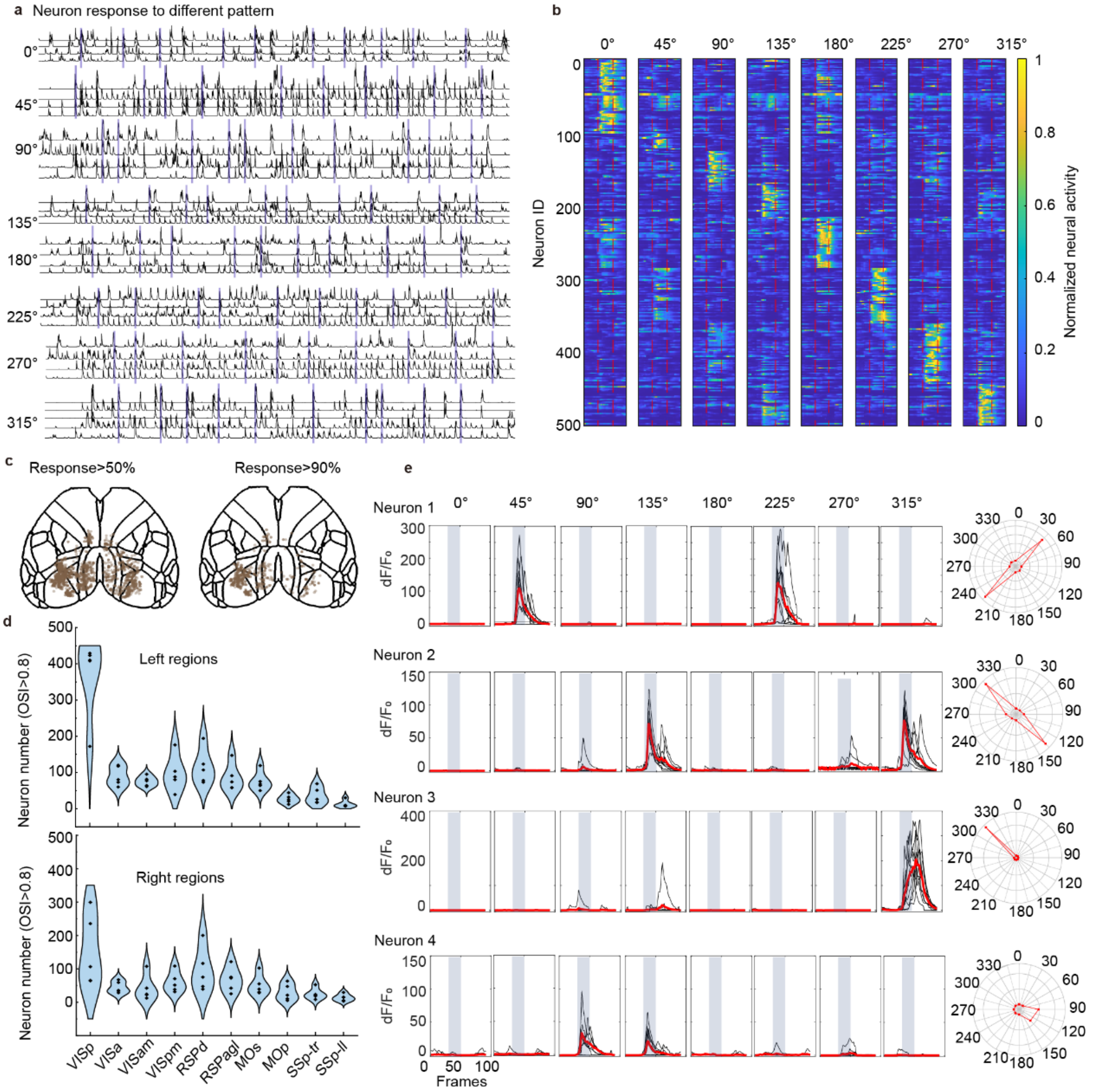

Supplementary Fig. 9 | Orientation specific neuronal responses a, Showcases examples of neural activity in response to different grating orientations, with the stimulation periods highlighted in blue boxes. **b**, The heatmap of neurons specifically respond to specific orientations. **c**, Maps the distribution of neurons responding to 50% and 90% of the stimuli trials. **d,** Violin plots of highly selective neurons: Compares the number of neurons with orientation selective index (OSI) > 0.8 across cortical regions of the left (top) and right (bottom) hemispheres, offering a hemispheric perspective on orientation selectivity (n=5 mice). **e,** The left panels display average neuronal responses to each orientation from example neurons, while the right panels feature polar plots illustrating the directional preferences of specific neurons.

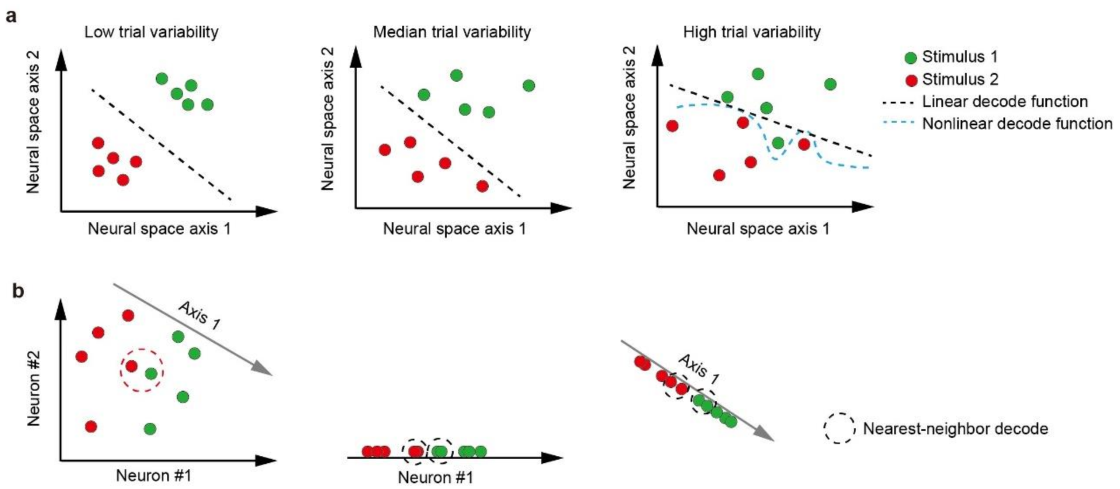

Supplementary Fig. 10 | Relationship between trial variability and coding ability. **a**, Diagram of increased trial-variability in neural space. Each dot is a single trial, different color stands for different stimulation type. Dashed lines are classification boundaries of either linear or nonlinear decoding methods. **b**, Diagram of decoding with nearest-neighbor read-out function.

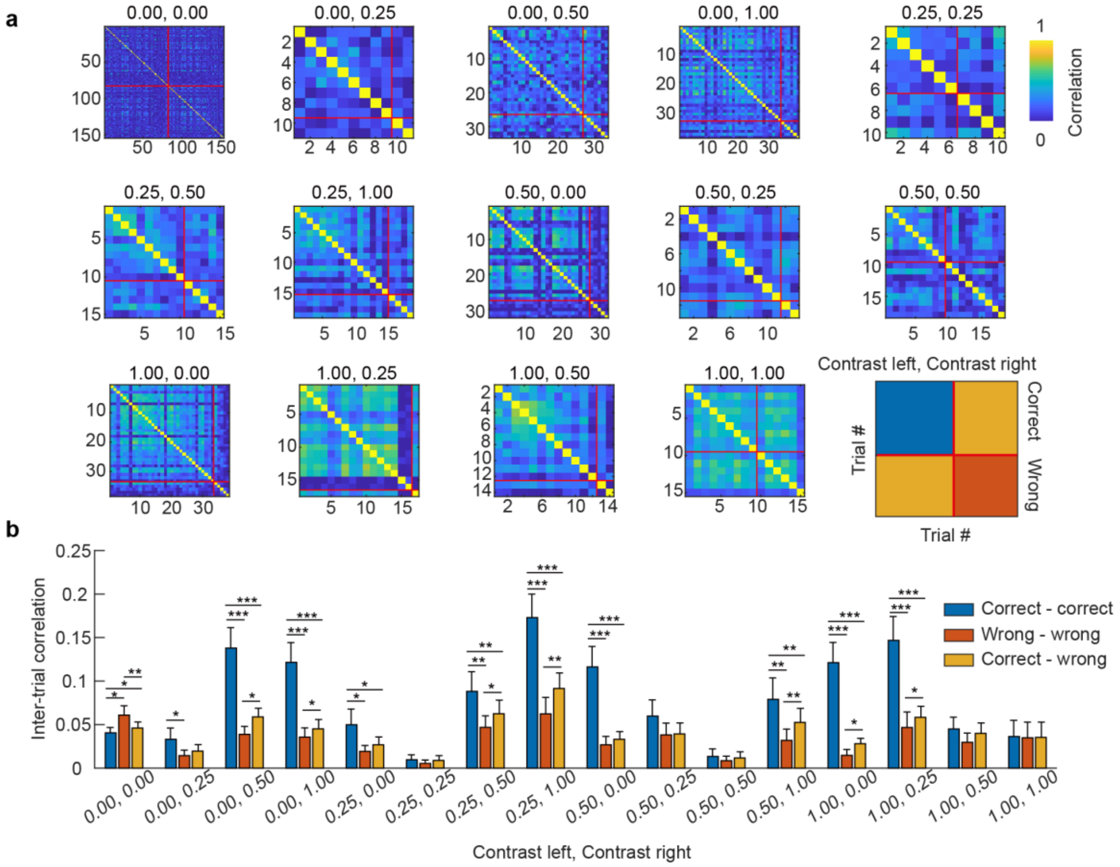

Supplementary Fig. 11 | Trial variability related to behavior performance. **a**, Inter-trial correlation matrix of an example session, with different cue types. Trials are sorted by the performance outcome. **b**, Mean inter-trials correlation grouped by the trial outcome and the cue contrast. Paired Wilcoxon signed rank test is used. *, $p<0.05$; **, $p<0.005$; ***, $p<0.0005$; error bar of SEM, n number is depended on the inter-trials number for each state.
