# Supplementary material for: Mesoscale neuronal granular trial variability in vivo illustrated by nonlinear recurrent network in silico
Source: Nat Commun. 2024 Nov 15;15:9894. doi: 10.1038/s41467-024-54346-3 (PMC11567969; doi:10.1038/s41467-024-54346-3)
Supplement: Supplementary file 2 — Description of Additional Supplementary Files [file 41467_2024_54346_MOESM2_ESM.pdf]

Description of Additional Supplementary Files

File name: Supplementary Movie 1
Description: Multi-modal signals synchronously recording in the head-fixed mouse. Simultaneous recording of multi-modal signals in a head-fixed mouse (Rasgrf2-2A-dCre/Ai148d). Facial expressions and wide-field cortical neural signals were recorded synchronously (left panel), while pupil area, running speed, and neural activity traces were analyzed simultaneously (right panel).

File name: Supplementary Movie 2
Description: Mesoscale imaging with high resolution and large field of view. Dynamic mesoscale imaging with high resolution across the wide cortex, and enlarged images from four selected regions.
